# To Keep Needles and Cutting Instruments Bright

**Published:** 1899-09

**Authors:** 


### To Keep Needles and Cutting Instruments Bright.

Dr. Dawburn recommends a saturated solution of washing soda in water as a cheap and reliable method of preserving the polish and edge of needles and cutting instruments. He has employed it upward of a year with complete satisfaction. Keeping them in alboline is also effectual, but the oiliness of the instruments is in a way objectionable. We may add that the former is actively antiseptic while the alboline is not.—*Annals of Surgery.*

